# Analytical Solution for Dynamic Response of a Reinforced Concrete Beam with Viscoelastic Bearings Subjected to Moving Loads

**DOI:** 10.3390/ma17184491

**Published:** 2024-09-13

**Authors:** Liangming Sun, Shuguang Liu, Fan Kong, Hanbing Zhao

**Affiliations:** 1 School of Civil Engineering and Architecture, Wuhan University of Technology, 122 Luoshi Road, Wuhan 430070, China; sunliangming@126.com (L.S.);; 2 Hubei Key Laboratory of Roadway Bridge and Structure Engineering, Wuhan University of Technology, 122 Luoshi Road, Wuhan 430070, China; 3 School of Civil Engineering, Hefei University of Technology, 193 Tunxi Road, Hefei 230009, China; 4 Centre for Infrastructure Engineering and Safety, School of Civil and Environmental Engineering, The University of New South Wales, Sydney, NSW 2052, Australia

**Keywords:** viscoelastic bearing RC beam, analytical solution, equidistant moving loads, dynamic response

## Abstract

To provide a theoretical basis for eliminating resonance and optimizing the design of viscoelastically supported bridges, this paper investigates the analytical solutions of train-induced vibrations in railway bridges with low-stiffness and high-damping rubber bearings. First, the shape function of the viscoelastic bearing reinforced concrete (RC) beam is derived for the dynamic response of the viscoelastic bearing RC beam subjected to a single moving load. Furthermore, based on the simplified shape function, the dynamic response of the viscoelastic bearing RC beam under equidistant moving loads is studied. The results show that the stiffness and damping effect on the dynamic response of the supports cannot be neglected. The support stiffness might adversely increase the dynamic response. Further, due to the effect of support damping, the free vibration response of RC beams in resonance may be significantly suppressed. Finally, when the moving loads leave the bridge, the displacement amplitude of the viscoelastic support beam in free vibration is significantly larger than that of the rigid support beam.

## 1. Introduction

**Figure 1:**
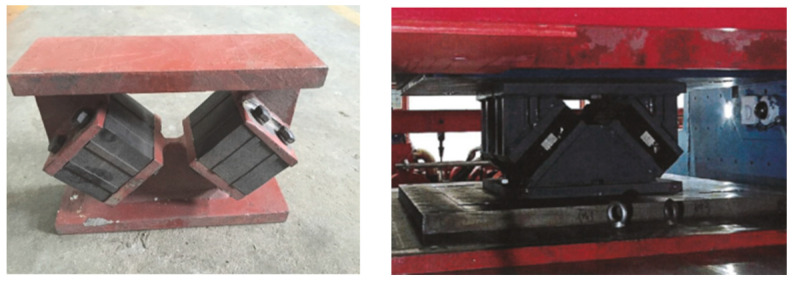
Test specimen of low-stiffness and high-damping rubber bearing.

The rail transit system plays an increasingly important role in public transportation. Trains traveling over bridges may cause harmful vibration of the underneath structure, inducing excessive noise pollution in the surrounding environment [1]. In this regard, structural engineers adopt different vibration control measures to mitigate the excessive vibration induced by trains. As an important component of connecting the superstructure and piers in bridge construction, a bridge bearing can effectively mitigate the train-induced vibration through the propagation route. At present, an engineering application shows that the low-stiffness and high-damping rubber bearing (Figure 1) for bridges developed by the authors’ research group [2] can isolate the train-induced vibration efficiently. However, understanding the vibration isolation mechanism of this viscoelastic rubber bearing for railway bridges from a theoretical perspective remains a significant challenge. Therefore, it is necessary to establish a dynamic model analytically for a viscoelastic bearing beam subjected to moving train loads. Moreover, developing a simplified analytical method for determining the dynamic response of the train–bridge system based on the established analytical model is of great importance, either from a theoretical or engineering perspective.

The vibration of the train–bridge system is an old and complicated problem. As early as 100 years ago, Stokes [3] and Willis [4] studied the damage mechanism of British Railway Bridges caused by strong vibration during train running. This was the first contribution to analyze the dynamic response of the train–bridge system with a rigid bearing. Since then, the theoretical computation approaches for determining the dynamic response of a rigidly supported beam subjected to moving train loads have been gradually improved. Ignoring the coupling function between train and bridge, Frýba [5] systematically studied the dynamic response of the train–bridge system. The analytical solutions of various train models, such as a moving load, moving mass, and so forth, were derived. Sudheesh Kumar et al. [6] investigated in detail the vibration cancellations of the bridges in free vibration under a single moving load. Wang et al. [7] studied the dynamic response of beams that are susceptible to large deformations under a single moving load. Moreover, Martínez-Rodrigo et al. [8], Xia et al. [9,10], and Xin et al. [11] considered the conditions of the simply supported bridge in resonance and cancellation.

For the train–bridge system with elastic bearings, some researchers including Yang et al. [12], Li et al. [13], Luo et al. [14], and Erduran et al. [15] used the finite element method or laboratory experiment method to analyze the vibration isolation effect of elastic bearings on the dynamic response of the train–bridge system and studied the effect of support stiffness on the vibration characteristics of a simply supported bridge. On the other hand, Yang et al. [16,17] analyzed the dynamic response of bridges with elastic bearings subjected to moving train loads, and further elucidated the mechanisms of resonance and vibration cancellation in the train-induced vibration of a bridge. Zhang et al. [18] and Zhao et al. [19] investigated the effectiveness of elastic bearings in reducing train-induced vibrations. Jiang et al. [20] derived the boundary condition equations based on the elastic bearings at both ends of a beam, and then obtained the expression of the main vibration mode based on the free vibration equation of the beam.

As far as the viscoelastic bearing beam is concerned, a recent literature review shows that very little attention has been focused on determining the dynamic response of a beam subjected to moving train loads. Interestingly, some researchers focus on the dynamic response of the considered beams subjected to impact load or explosion load. For example, Chen et al. [21] proposed a method for calculating the elastic dynamic response of reinforced concrete beams with a viscoelastic bearing under impact loads based on the theory of Euler–Bernoulli beams and the quasi-static Hertz contact theory. Song et al. [22] proposed both elastic and plastic analytical methods for bridges with flexible supports and restraints under blast loads. Çalım [23] analyzed the dynamic response of beams on viscoelastic foundations subjected to impact loads based on the Timoshenko beam theory. A viscoelastic bearing in the train–bridge system cannot be modeled like one in elastic/rigid bearing beams. To summarize, a further analytical investigation of the effect of a viscoelastic bearing on the dynamic response of beams under moving train loads is of significant importance.

Based on the previous vibration theories of rigid support beams and elastic support beams, this paper derives the dynamic response of viscoelastic support beams under moving loads. This work provides theoretical support for the potential large-scale application of viscoelastic bearings in the future and enriches the dynamic calculation methods for train–bridge systems. Specifically, the shape function of the viscoelastic bearing beam is derived from the dynamic response of the viscoelastic bearing beam under free vibration. Then, based on the obtained shape function, the dynamic response of the viscoelastic-supported beam under a single moving load is derived using Duhamel’s integral. Furthermore, this paper simplifies the shape function by equating it to the superposition of the mode shape functions of a viscoelastic-supported rigid beam and an elastic beam with rigid supports. Based on this simplified shape function, the dynamic response of viscoelastic-supported beams under the action of equidistant moving loads is derived. In the future, this article can provide theoretical support for the vibration reduction of viscoelastic bearings in practical engineering.

## 2. Mathematical Model of the Viscoelastic Bearing RC Beam

### 2.1. Modal Shape of Vibration

**Figure 2:**
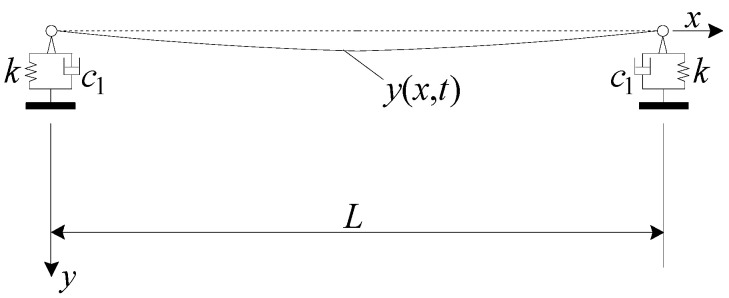
Simplified calculation model of a viscoelastic bearing RC beam.

The simplified calculation model of an RC beam for a railway line is shown in Figure 2, where two identical viscoelastic bearings with vertical stiffness k and damping coefficient $c_{1}$ support a track girder with the mass per unit length $\bar{m}$. For deriving the closed-form solution of the track girder shape function, the Euler–Bernoulli beam assumption is adopted [24]. Assume further that the mass of the bearings is negligible compared with that of the bridge. In addition, the damping of high-speed railway box girders (ξ < 0.05) can generally be ignored, and the time for a train to pass over the bridge is extremely short. As a result, the energy dissipated by damping is limited with a minimal impact on the dynamic response of the bridge [25]. Therefore, ignoring its damping also has high practicality in engineering [25]. The girder length is *L*, and the girder’s flexural rigidity is EI, with E as the Young’s modulus and I the cross-sectional moment of inertia. The vertical free vibration equation for the displacement *y*(*x*, *t*) of the RC beam is determined by a partial differential equation [24], as shown in Equation (1).

(1) $$EI\frac{\partial^{4}y\left(x,t\right)}{\partial x^{4}}+\bar{m}\frac{\partial^{2}y\left(x,t\right)}{\partial t^{2}}=0$$

where t is the time parameter; x denotes the horizontal coordinate.

Following the method of separation of variables, the vertical displacement *y*(*x*, *t*) of the viscoelastic bearing RC beam can be expressed by separation of variables [24]. Thus

(2) $$y\left(x,t\right)=T\left(t\right)X\left(x\right)\;$$

where *T*(*t*) denotes the time-dependent function, and *X*(*x*) denotes the shape function of vibration. Thus, Equation (1) can be rewritten as

(3) $$\frac{1}{X(x)}\frac{d^{4}X(x)}{dx^{4}}=-\frac{1}{T(t)}\frac{\bar{m}}{EI}\frac{d^{2}T(t)}{dt^{2}}=\lambda^{4}$$

Equation (3) further yields

(4) $$\frac{d^{4}X(x)}{dx^{4}}-\lambda^{4}X(x)=0$$

(5) $$\frac{d^{2}T(t)}{dt^{2}}-\omega^{2}T(t)=0$$

where

(6) $$\lambda^{2}=\omega \sqrt{\frac{\bar{m}}{EI}}$$

ω is the natural frequency of the RC beam with a viscoelastic bearing; *λ* is a constant, which can be obtained from the natural frequency *ω*, stiffness *EI*, and mass of the structure $\bar{m}$ [24]. The general solution to the second equation in Equation (5) is written as

(7) $$T(t)=A_{1}\sin\left(\omega t\right)+A_{2}\cos\left(\omega t\right)$$

where *A*_1_ and *A*_2_ are unknown constants depending on the initial conditions:

(8) $$\underset{t\to 0}{\lim}\;y(x,t)=0$$

(9) $$\underset{t\to 0}{\lim}\frac{dy(x,t)}{dt}=0$$

The general solution to the first equation in Equation (4) is written as

(10) X(x)=aH(λx)

where $H\left(\lambda x\right)={\left[sin(\lambda x),cos(\lambda x),\sin(\lambda x),\cosh(\lambda x)\right]}^{T}$; $a=[a_{1},a_{2},a_{3},a_{4}]$ are four unknown constants depending on the boundary conditions (the bending moments at both ends of a simply supported beam are zero. The shear forces at both ends depend on the stiffness and damping of the viscoelastic support bearings, as well as the vertical displacement and velocity at the ends of the beam).

(11) $${\left.M\right|}_{x=0}={\left.EI\frac{d^{2}y}{dx^{2}}\right|}_{x=0}=0$$

(12) $${\left.Q\right|}_{x=0}={\left.EI\frac{d^{3}y}{dx^{3}}\right|}_{x=0}={\left.-ky\right|}_{x=0}-{\left.c_{1}\frac{dy}{dt}\right|}_{x=0}$$

(13) $${\left.M\right|}_{x=L}={\left.EI\frac{d^{2}y}{dx^{2}}\right|}_{x=L}=0$$

(14) $${\left.Q\right|}_{x=L}={\left.EI\frac{d^{3}y}{dx^{3}}\right|}_{x=L}={\left.ky\right|}_{x=L}+{\left.c_{1}\frac{dy}{dt}\right|}_{x=L}$$

Combining Equation (2) with Equations (10)–(14) yields

(15) $$(a_{4}-a_{2})T(t)=0$$

(16) $$\lambda^{3}EI\left(a_{3}-a_{1}\right)T(t)=-[kT(t)+c_{1}T^{\prime }(t)]\left(a_{2}+a_{4}\right)$$

(17) $$EIT(t)aH_{1}(\lambda L)=0$$

(18) $$\lambda^{3}EIT(t)aH_{2}(\lambda L)=[kT(t)+c_{1}T^{\prime }(t)]aH(\lambda L)$$

where

$$H_{1}\left(\lambda L\right)={\left[-sin(\lambda L),-cos(\lambda L),\sinh(\lambda L),\cosh(\lambda L)\right]}^{T},$$

$$H_{2}\left(\lambda L\right)={\left[-\cos(\lambda L),sin(\lambda L),cosh(\lambda L),sinh(\lambda L)\right]}^{T}.$$

Introducing two variables *G*_1_ and *G*_2_

(19) $$G_{1}=\frac{k}{\lambda^{3}EI},G_{2}=\frac{c_{1}}{\lambda^{3}EI}$$

and considering zero initial conditions (the displacement and velocity of the beam are both 0 at the initial stage) shown in Equations (8) and (9) leads to

(20) $$-\sin\left(\lambda L\right)a_{1}+[\cosh\left(\lambda L\right)-\cos\left(\lambda L\right)]a_{2}+\sinh\left(\lambda L\right)a_{3}=0$$

(21) $$-a_{1}+2(G_{1}+G_{2})a_{2}+a_{3}=0$$

(22) $$\begin{array}{l} {}[-\cos\left(\lambda L\right)-(G_{1}+G_{2})\sin\left(\lambda L\right)]a_{1}+\\ \{\sin\left(\lambda L\right)+\sinh\left(\lambda L\right)-(G_{1}+G_{2})[\cos\left(\lambda L\right)+\cosh\left(\lambda L\right)]\}a_{2}+\\ {}[\cosh\left(\lambda L\right)-(G_{1}+G_{2})\sinh\left(\lambda L\right)]a_{3}=0 \end{array}$$

Given that homogeneous Equations (20)–(22) exist in a non-zero solution, the determinant value of the coefficients must be zero. The corresponding natural vibration equation can be obtained as

(23) $$[1-\cos(\lambda L)\cosh(\lambda L)]+2{\left(G_{1}+G_{2}\right)}^{2}\sin(\lambda L)\sinh(\lambda L)+2(G_{1}+G_{2})|H_{3}(\lambda L)|=0.$$

where

$$H_{3}(\lambda L)=\left[\begin{array}{cc} \cos(\lambda L) & \cosh(\lambda L)\\ \sin(\lambda L) & \sinh(\lambda L) \end{array}\right].$$

After the structural parameters other than *λ* are determined, the graph of the function on the left-hand side of the equation can be plotted using MATLAB R2021b to roughly determine the interval of its zeros. Then, the numerical solution for *λ* in Equation (23) can be obtained through the bisection method. Similarly, by Equations (20)–(22), the coefficients *a*_2_, *a*_3_, *a*_4_ can be determined as

(24) $$a_{2}=a_{4}=\frac{n_{1}}{d}a_{1}$$

(25) $$a_{3}=\frac{n_{2}}{d}a_{1}$$

where

$$n_{1}=\sinh\left(\lambda L\right)-\sin\left(\lambda L\right),$$

$$n_{2}=2(G_{1}+G_{2})\sin\left(\lambda L\right)-[\cosh\left(\lambda L\right)-\cos\left(\lambda L\right)],$$

$$d=2(G_{1}+G_{2})\sinh\left(\lambda L\right)-[\cosh\left(\lambda L\right)-\cos\left(\lambda L\right)].$$

Combining Equation (10), (24) and (25) yields the shape function of the RC beam:

(26) $$X\left(x\right)=\sin\left(\lambda x\right)+\left\{\frac{n_{1}}{d}[\cos\left(\lambda x\right)+\cosh\left(\lambda x\right)]\right.+\left.\frac{n_{2}}{d}\sinh\left(\lambda x\right)\right\}$$

Here, the shape function of the RC beam with a viscoelastic bearing can be regarded as the superposition of the flexural deflection modal shape of the simply supported beam (the first term), and the influence function is controlled by bearing stiffness and bearing damping on the displacement of the RC beam (the second term).

### 2.2. Analytical Solution for Dynamic Response of Viscoelastic Bearing RC Beam

**Figure 3:**
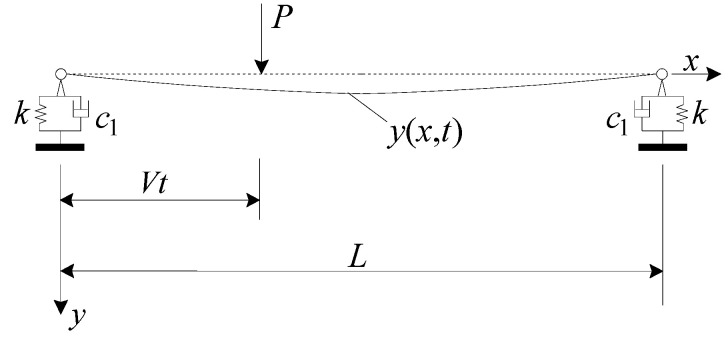
Simplified calculation model of viscoelastic bearing RC beam subjected to a moving load.

For an RC beam with a viscoelastic bearing subjected to a single moving load *P* of speed *V*, as indicated in Figure 3, the equation of motion for the displacement *y*(*x*, *t*) of the RC beam is [25]

(27) $$EI\frac{\partial^{4}y(x,t)}{\partial x^{4}}+\bar{m}\frac{\partial^{2}y(x,t)}{\partial t^{2}}+c\frac{\partial y(x,t)}{\partial t}=\delta (x-Vt)P$$

where $\bar{m}$ is the mass per unit length of the RC beam, *EI* is the flexural rigidity of the RC beam, *c* is the damping coefficient of the RC beam, and *δ* is the Dirac delta function. According to the concept of modal superposition, the vertical displacement *y*(*x*, *t*) of the viscoelastic bearing RC beam can be expressed [25]. Thus

(28) $$y\left(x,t\right)=\sum_{i=1}^{\infty }X_{i}\left(x\right)q_{i}\left(t\right)$$

where $X_{i}\left(x\right)$ denotes the shape function of the *i*-th mode, and $q_{i}\left(t\right)$ is the generalized coordinate of the *i*-th mode. Note that the RC beam subjected to moving loads is a transient problem with short acting time. In this case, considering the first mode only and neglecting other higher modes still yields a satisfactory solution. Specifically, multiplying both sides of Equation (27) by the shape of function Equation (26) and integrating with respect to the length *L* of the RC beam leads to

(29) $${\ddot{q}}_{1}(t)+2\xi_{1}\omega_{1}{\dot{q}}_{1}(t)+\omega_{1}^{2}q_{1}(t)=\frac{1}{\bar{m}}\frac{1}{\int_{0}^{L}{\left[X_{1}\left(x\right)\right]}^{2}dx}PX_{1}\left(Vt\right)$$

where $\xi_{1}$ represents the damping ratio of the RC beam–viscoelastic bearing system, $\bar{m}$ is the uniformly distributed mass of the RC beam, P denotes the load magnitude, *V* is the moving speed of the load, and *t* is time. The fundamental frequency of RC beam $\omega_{1}$ and the shape function $X_{1}\left(x\right)$ of the viscoelastic bearing RC beam can be obtained from *λ*, as shown in Section 2.1. Considering Rayleigh’s method gives the damping ratio *ξ*_1_ of the viscoelastic bearing RC beam:

(30) $$\xi_{1}=\xi_{b}+\frac{c_{1}\left\{{\left[X_{1}\left(0\right)\right]}^{2}+{\left[X_{1}\left(L\right)\right]}^{2}\right\}}{2\omega_{1}\bar{m}\int_{0}^{L}{\left[X_{1}\left(x\right)\right]}^{2}dx}$$

where $\xi_{1}$ can be regarded as the superposition of the damping ratio of the simply supported beam $\xi_{b}$ (the first item) and the effect of bearing damping on the RC beam (the second item).

Applying Duhamel’s integral [25] to Equation (29) yields the generalized coordinate *q*_1_(*t*):

(31) $$q_{1}\left(t\right)=\frac{1}{\bar{m}\omega_{D}}\frac{P}{\int_{0}^{L}{\left[X_{1}\left(x\right)\right]}^{2}dx}\int_{0}^{t}X_{1}\left(V\tau \right)\sin\omega_{D}\left(t-\tau \right)\exp\left[-\xi_{1}\omega_{1}\left(t-\tau \right)\right]d\tau$$

where $\omega_{D}=\omega_{1}\sqrt{1-\xi_{1}^{2}}$. Thus, the vertical displacement *y*(*x*, *t*) of the viscoelastic bearing RC beam can be written as

(32) $$y\left(x,t\right)=X_{1}\left(x\right)q_{1}\left(t\right)$$

Equation (32) is the displacement response of a viscoelastic bearing RC beam subjected to a single moving load in forced vibration. However, after the load leaves the RC beam, the system free vibration can be written as

(33) $$y\left(x,t\right)=[y\left(x,L/v\right)\cos\left(\omega_{D}t\right)+\frac{\dot{y}\left(x,L/v\right)+y\left(x,L/v\right)\xi_{1}\omega_{1}}{\omega_{D}}\sin\left(\omega_{D}t\right)]\times \exp\left(-\xi_{1}\omega_{1}t\right)$$

where $y\left(x,L/v\right)$ and $\dot{y}\left(x,L/v\right)$ denote the displacement and velocity of the first modal shape at t=L/V, respectively, which are the initial conditions of the free vibration and can be derived from Equation (32).

### 2.3. Simplification of the Modal Shape

**Figure 4:**
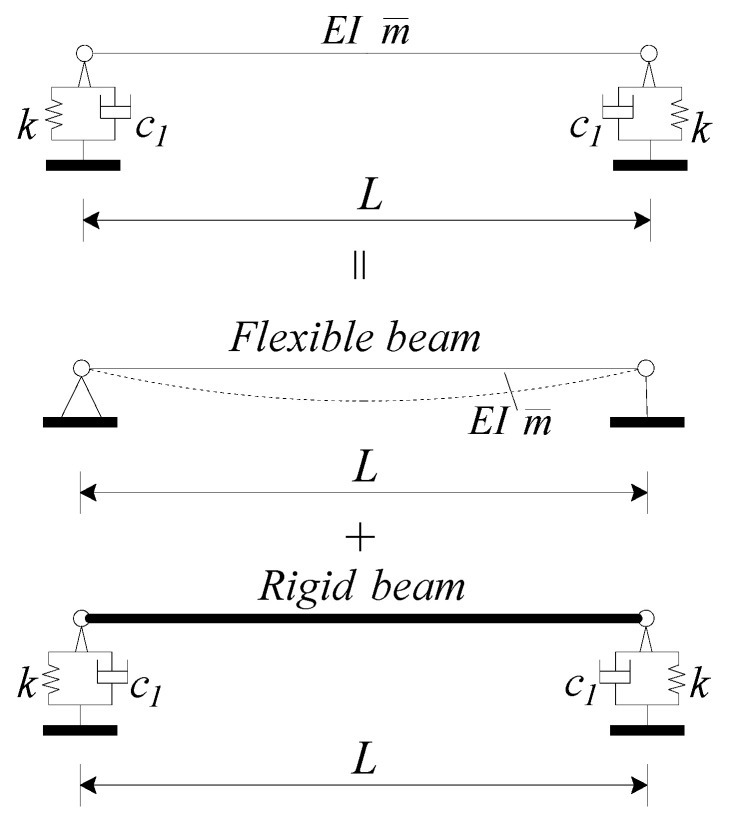
Simplified mode shape function of viscoelastic bearing RC beam.

As shown in Equation (26), the shape function is a complex trigonometric function expression. In practical engineering, the loads are often more complicated. For instance, in Section 2.5, the train is simplified as an equidistant moving load. To enhance the practicality of the shape function $X\left(x\right)$, further simplification is required. The simplification method is illustrated in Figure 4.

Assume the shape function of the viscoelastic bearing RC beam can be approximated as the superposition of two terms. They are the first flexural deflection modal shape of the simply supported beam and the influence function of the displacement of the rigid beam by bearing stiffness and bearing damping, as indicated in Figure 4. Thus, the shape function of the viscoelastic bearing RC beam can be obtained approximately:

(34) $$\varphi \left(x\right)=\sin(\frac{\pi x}{L})+\kappa$$

Considering the boundary conditions of the viscoelastic bearing RC beam, the sum of elastic force and damping force at the support is equal to the shear force at the support of the RC beam. Thus, combining Equation (11) with Equation (34) yields

(35) $$\varphi \left(x\right)=\sin(\frac{\pi x}{L})+\kappa =\sin(\frac{\pi x}{L})+\frac{EI\pi^{3}}{L^{3}(k+c_{1})}$$

As can be seen from Equation (35), when the bearing stiffness *k* is infinite, and the bearing damping *c*_1_ is zero, the mode function *φ*(*x*) reduces to the first modal shape of the simply supported beam. Moreover, when the bearing damping *c*_1_ is zero, the mode function *φ*(*x*) reduces to the first mode shape of the elastic bearing beam proposed in [20].

Thus, the vertical displacement *y*(*x*, *t*) of the viscoelastic bearing RC beam can be expressed as

(36) $$y\left(x,t\right)=\varphi \left(x\right)q\left(t\right)=[\sin(\frac{\pi x}{L})+\kappa ]\cdot q(t)=[\sin(\frac{\pi x}{L})+\frac{EI\pi^{3}}{L^{3}(k+c_{1})}]\cdot q(t)$$

### 2.4. Approximate Analytical Solution for the Viscoelastic Bearing RC Beam Subjected to a Single Moving Load

Combining Equation (29) with Equation (36) yields an approximate dynamic equation of the viscoelastic bearing RC beam subjected to a single moving load:

(37) $$\ddot{q}(t)+2\xi \omega \dot{q}(t)+\omega^{2}q(t)=\frac{2\pi P}{\bar{m}L\left(2\kappa^{2}\pi +8\kappa +\pi \right)}\left[\sin(\frac{\pi V}{L}t)+\kappa \right]$$

where the fundamental frequency ω can be obtained by Rayleigh’s method; the damping ratio ξ can be solved from Equation (30).

(38) $$\omega^{2}=\frac{\int_{0}^{L}EI{\left[\varphi^{\prime \prime }\left(x\right)\right]}^{2}dx+k\left\{{\left[\varphi \left(0\right)\right]}^{2}+{\left[\varphi \left(L\right)\right]}^{2}\right\}}{\int_{0}^{L}\bar{m}{\left[\varphi \left(x\right)\right]}^{2}dx}$$

Similarly, applying Duhamel’s integral [25] to Equation (37) and considering Equation (32) leads to

(39) $$y\left(x,t\right)=\left[sin\left(\frac{\pi x}{L}\right)+\kappa \right]\frac{2\pi P}{\bar{m}\omega^{2}L\left(2\kappa^{2}\pi +8\kappa +\pi \right)}\left\{\left[\frac{1}{{\left(1-\beta^{2}\right)}^{2}+{\left(2\xi \beta \right)}^{2}}\right]\left(Q_{1}+Q_{3}\right)+\kappa Q_{2}\right\}$$

where

(40) $$\begin{array}{l} Q_{1}=\left[\left(1-\beta^{2}\right)\sin\left(\bar{\omega }t\right)-2\xi \beta \cos\left(\bar{\omega }t\right)\right],\\ Q_{2}=1-\exp\left(-\xi \omega t\right)[\cos\left(\omega_{D}t\right)+\xi \sqrt{1-\xi^{2}}\sin\left(\omega_{D}t\right)],\\ Q_{3}=\exp\left(-\xi \omega t\right)[2\xi \beta \cos\left(\omega_{D}t\right)-\frac{\beta \left(1-\beta^{2}-2\xi^{2}\right)}{\sqrt{1-\xi^{2}}}\sin\left(\omega_{D}t\right)] \end{array}$$

where $\bar{\omega }=\pi V/L$ denotes the driving frequency implied by the moving loads, and $\beta =\bar{\omega }/\omega$ denotes the speed parameter.

### 2.5. Approximate Analytical Solution for the Viscoelastic Bearing RC Beam Subjected to Equidistant Moving Loads

**Figure 5:**
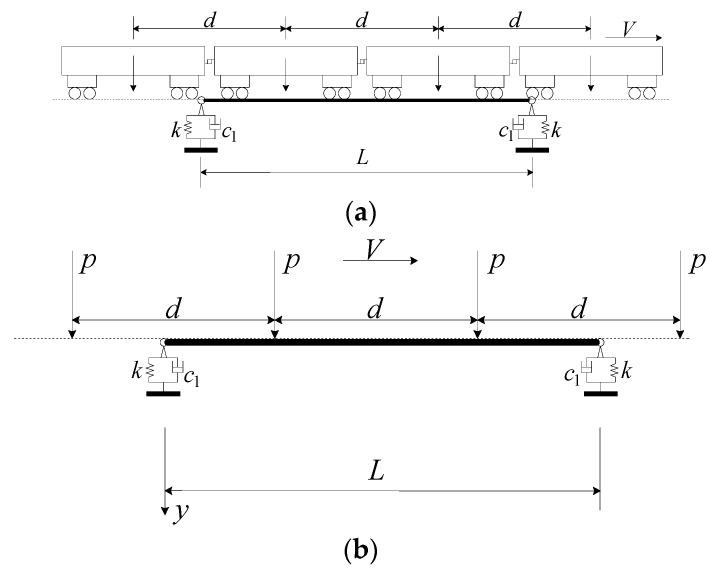
Calculation model: (**a**) model of train; (**b**) simplified calculation model of viscoelastic bearing RC beam subjected to equidistant moving loads.

In practical engineering, trains are primarily composed of multiple carriages connected in series. The dynamic analysis of vehicle–bridge coupling in practical engineering is extremely complex, often involving numerous random factors (such as wheel irregularity, geometric and dynamic track irregularity, and the snake-like motion of wheelsets, etc.). Due to the limitations of computational methods, highly simplified models have to be adopted, for instance, simplifying the train load into a uniformly distributed moving load, as shown in Figure 5. Each carriage can be equivalently represented as a concentrated force acting at the center of the carriage.

The analytical approach of the viscoelastic bearing RC beam subjected to a series of concentrated loads *P* of equal intervals *d* at speed *V* can be expressed from Equation (36). Substituting Equation (36) into Equation (27), multiplying both sides of the equation by the shape function *φ*(*x*), and then integrating with respect to the length *L* of the RC beam yields

(41) $$\ddot{q}(t)+2\xi \omega \dot{q}(t)+\omega^{2}q(t)=\frac{2\pi P_{k}}{\bar{m}L\left(2\kappa^{2}\pi +8\kappa +\pi \right)}$$

where

(42) $$\begin{array}{ll} P_{k} & =\sum_{k=1}^{N}P\left[\sin\left(\frac{\pi V}{L}\left(t-t_{k}\right)\right)+\kappa \right]\left[H\left(t-t_{k}\right)-H\left(t-t_{k}-\frac{L}{V}\right)\right]\\ & =P\sum_{k=1}^{N}\left\{\left[\sin\left(\frac{\pi V}{L}\left(t-t_{k}\right)\right)+\kappa \right]H\left(t-t_{k}\right)+\left[\sin\left(\frac{\pi V}{L}\left(t-t_{k}-\frac{L}{V}\right)\right)-\kappa \right]H\left(t-t_{k}-\frac{L}{V}\right)\right\} \end{array}$$

In Equation (42), $t_{k}=\left(k-1\right)d/V$ denotes the arrival time of the *k*th load on the RC beam; $H\left(\cdot \right)$ is the Heaviside function. Thus, the Heaviside function can be used to consider whether the load is on the RC beam at each moment. That is,

(43) $$H\left(t-t_{k}\right)-H\left(t-t_{k}-\frac{L}{V}\right)=\left\{\begin{array}{cc} 0 & t<t_{k},\\ 1 & t_{k}\leq t\leq t_{k}+L/V,\\ 0 & t>t_{k}+L/V \end{array}\right.$$

The displacement response of the viscoelastic bearing RC beam subjected to equidistant moving loads can be solved as

(44) $$\begin{array}{ll} y\left(x,t\right)= & \frac{2\pi P}{\bar{m}\omega^{2}L\left(2\kappa^{2}\pi +8\kappa +\pi \right)}[\sin(\frac{\pi x}{L})+\kappa ]\{\sum_{k=1}^{N}[S_{1}(t-t_{k})H(t-t_{k})+\\ & S_{1}(t-t_{k}-L/V)H(t-t_{k}-L/V)]+\kappa \sum_{k=1}^{N}[S_{2}(t-t_{k})H(t-t_{k})-\\ & \;\;\;\;\;\;\;\;\;S_{2}(t-t_{k}-L/V)H(t-t_{k}-L/V)]\} \end{array}$$

where

(45) $$\begin{array}{ll} S_{1}\left(t\right) & =\frac{1}{{\left(1-\beta^{2}\right)}^{2}+{\left(2\xi \beta \right)}^{2}}\{(1-\beta^{2})\sin(\bar{\omega }t)-2\xi \beta \cos(\bar{\omega }t)+\\ & \;\exp(-\xi \omega t)[2\xi \beta \cos(\omega_{D}t)-\frac{\beta (1-\beta^{2}-2\xi^{2})}{\sqrt{1-\xi^{2}}}\sin\left(\omega_{D}t\right)]\},\\ S_{2}\left(t\right) & =1-\exp(-\xi \omega t)[\cos(\omega_{D}t)+\frac{\xi }{\sqrt{1-\xi^{2}}}\sin(\omega_{D}t)] \end{array}$$

## 3. Numerical Examples

### 3.1. Validation of Analytical Solution: Degenerate Case

A simply supported RC beam in [25] is taken as an illustrative example to validate the analytical solution of the viscoelastic bearing. Some parameters of the RC beam are shown in Table 1. Moreover, a single moving load *P* = 840 kN with constant speed (*V* = 40 m/s) is considered.

The viscoelastic bearing RC beam degenerates to a rigid bearing RC beam when the stiffness of the supports is infinite (*k* = +∞) and the damping of the supports is zero (*c*_1_ = 0). The first four eigenvalues obtained from Equation (23) are 0.0981, 0.1963, 0.2945, and 0.3927. To simplify the calculation, only the first modal shape of the beam is considered. Applying the rule of L’Hospital to Equation (26) yields a simplified expression of the shape function *X*(*x*). That is,

(46) $$X\left(x\right)=\sin\left(\lambda x\right)+\frac{\sinh\left(\lambda x\right)\sin\left(\lambda L\right)}{\sinh\left(\lambda L\right)}$$

**Figure 6:**
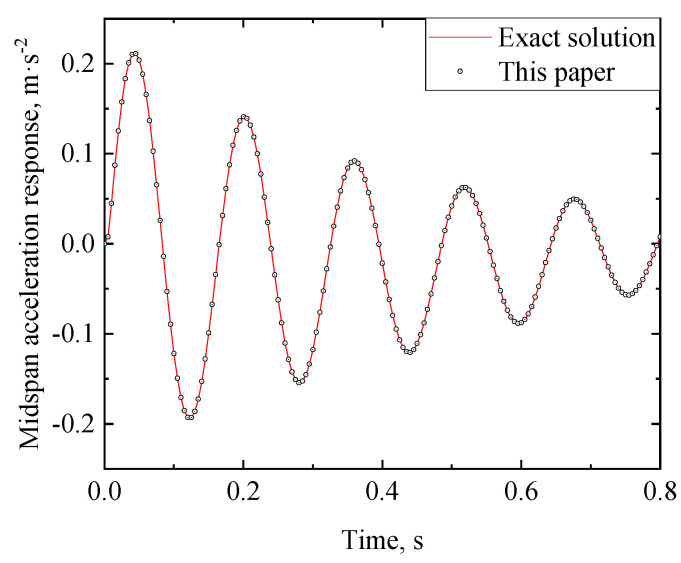
Midspan acceleration time history of the RC beam in the degenerated case of k=∞ and $c_{1}=0$.

**Figure 7:**
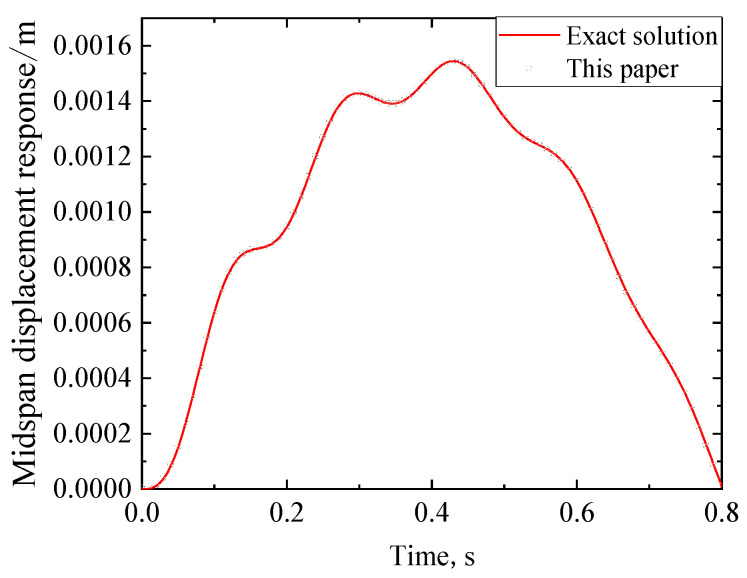
Midspan displacement time history of the RC beam in the degenerated case of k=∞ and $c_{1}=0$.

The displacement response of the RC beam can be obtained from Equations (29)–(32). Figure 5 and Figure 6 show the midspan acceleration and displacement of the RC beam, respectively, where the analytical solution of the dynamic response of the rigid bearing RC beam [25] is also plotted for comparison. When the bearing stiffness *k* and damping *c*_1_ are taken as +∞ and 0, respectively, the analytical solution of the viscoelastic bearing beam under moving load degrades to that of a rigid bearing beam (the latter can be regarded as an extreme case of the viscoelastic bearing beam). As shown in Figure 6 and Figure 7, the dynamic response of the viscoelastic bearing beam in this case is in good agreement with the existing dynamic response of the rigidly supported beam, which proves the validity of the analytical method proposed in this paper.

### 3.2. Accurate Solution for Viscoelastic Bearing RC Beam Subjected to a Single Moving Load

#### 3.2.1. Response of the Viscoelastic Bearing RC Beam under Typical Parameters

**Figure 8:**
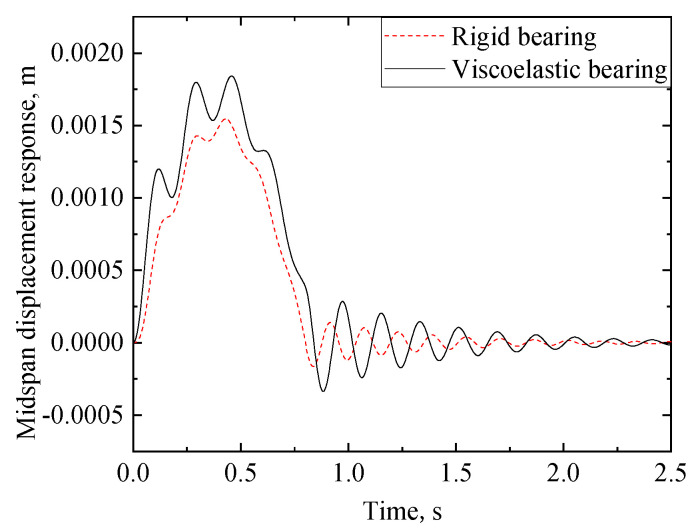
Midspan displacement time history of the RC beam with different bearing conditions.

**Figure 9:**
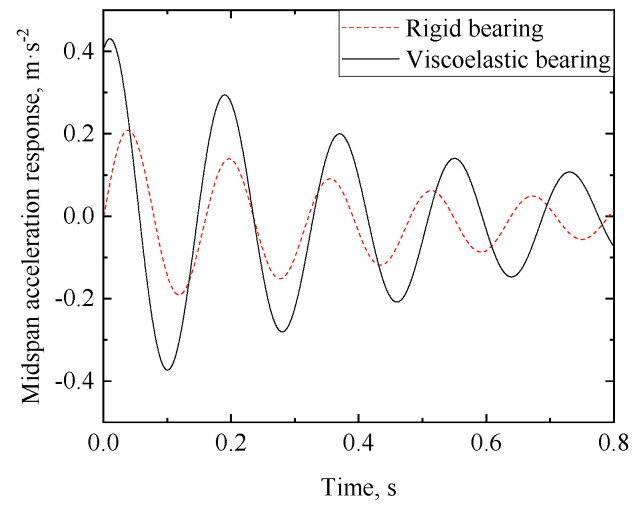
Midspan acceleration time history of the RC beam with different bearing conditions.

Choose the stiffness of the supports *k* = 1600 kN/mm and the damping of the supports *c*_1_ = 1200 (kN·s)/m; the other parameters are referred to in Section 3.1. In this case, the first mode eigenvalue *λ*_1_ = 0.0092 is solved from Equation (23). The displacement response of the viscoelastic bearing RC beam can be solved from Equations (29)–(33). Figure 8 and Figure 9 show the dynamic response of the rigid bearing RC beam and that of the viscoelastic bearing RC beam. From Figure 8 and Figure 9, one can see that the RC beam is in forced vibration during 0 < *t* ≤ 0.8 s. After the load leaves the RC beam at 0.8 s, the effect of the stiffness and damping of the supports on the response cannot be neglected. Moreover, due to the effect of the infinite support stiffness, the response amplitude and the peak time of the viscoelastic bearing RC beam are greater than those of the rigid bearing RC beam. Furthermore, due to the effect of the damping of the supports, the response amplitude of the viscoelastic bearing RC beam decays faster than that of the rigid bearing RC beam. 

#### 3.2.2. Effect of Train Speed

**Figure 10:**
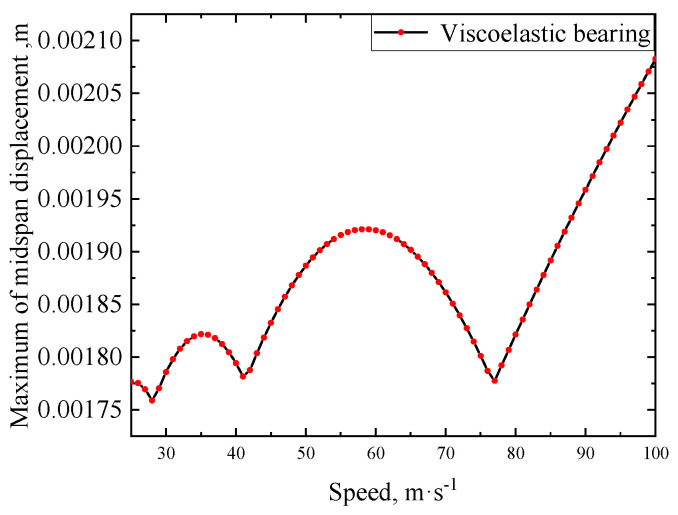
Comparison of midspan displacements of viscoelastic bearing RC beam under different train speeds.

Following the same procedures utilized for generating Figure 8, the displacement peak responses of the viscoelastic bearing RC beam under different train speeds are calculated and shown in Figure 10. The other parameters referred to are those used for Figure 8. As can be seen from Figure 10, the maximum displacement response does not increase linearly with the increase in train speed. The speeds corresponding to peak responses are 35 m/s or 59 m/s, commonly defined as the resonance speeds.

#### 3.2.3. Effect of Stiffness at the Supports

**Figure 11:**
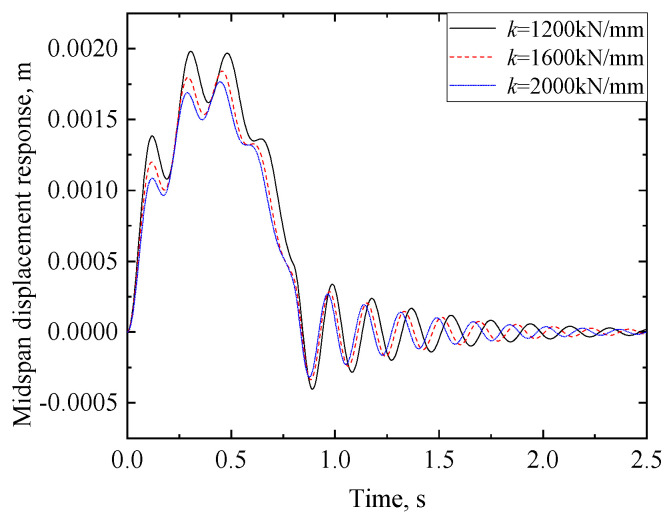
Comparison of midspan displacements of viscoelastic bearing RC beam with different stiffnesses at the supports.

Following the same procedures utilized for generating Figure 8, the midspan displacement responses of the viscoelastic bearing RC beam with different stiffnesses of the supports are shown in Figure 11. The other parameters referred to are those used for Figure 8. As shown in Figure 11, the maximum displacement of the viscoelastic bearing RC beam decreases with the increasing support stiffness. Specifically, when the support stiffness *k* is 1200 kN/mm, 1600 kN/mm, and 2000 kN/mm, the maximum displacement response is 1.98 mm, 1.84 mm, and 1.78 mm, respectively. Further, the peak time also increases slightly with decreasing support stiffness, reflecting the effect of the support stiffness on the natural frequency of the RC beam. The natural frequency of the RC beam also increases with increasing support stiffness.

#### 3.2.4. Effect of Damping at the Supports

**Figure 12:**
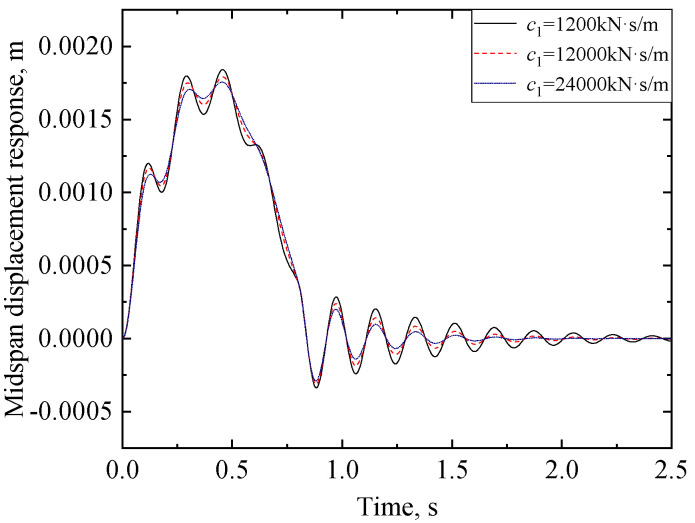
Comparison of midspan displacement of viscoelastic bearing RC beam with different damping at the supports.

We analyze next the effect of the damping of the supports on the displacement response of the viscoelastic bearing RC beam. The midspan displacement responses of the viscoelastic bearing RC beam with different bearing damping coefficients are shown in Figure 12; the other parameters used in this example are those used for Figure 8. The following are some comments regarding the effect of the support damping on the midspan displacement. From Figure 12, it seems that the midspan displacement of the viscoelastic bearing RC beam decreases slightly with the increasing support damping. Specifically, first, for the cases with support damping *c*_1_ = 1200 kN·s/m, *c*_1_ = 12,000 kN·s/m, and *c*_1_ = 24,000 kN·s/m, the maximum displacement response at the midspan is 1.84 mm, 1.79 mm, and 1.75 mm, respectively. Further, during the free vibration phase, the decaying rate of the displacement with respect to time increases with increasing support damping. Finally, the speeds corresponding to peak responses with the different damping of the supports are identical, reflecting that the girder’s natural frequency is almost unaffected by the support damping.

### 3.3. Approximate Solution for the Viscoelastic Bearing RC Beam Subjected to a Single Moving Load

**Figure 13:**
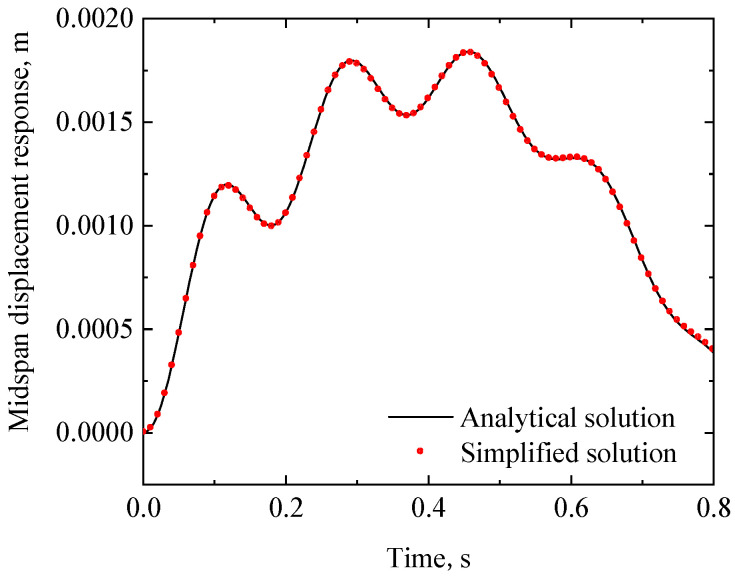
Curves of displacement versus time for midspan of viscoelastic bearing RC beam.

**Figure 14:**
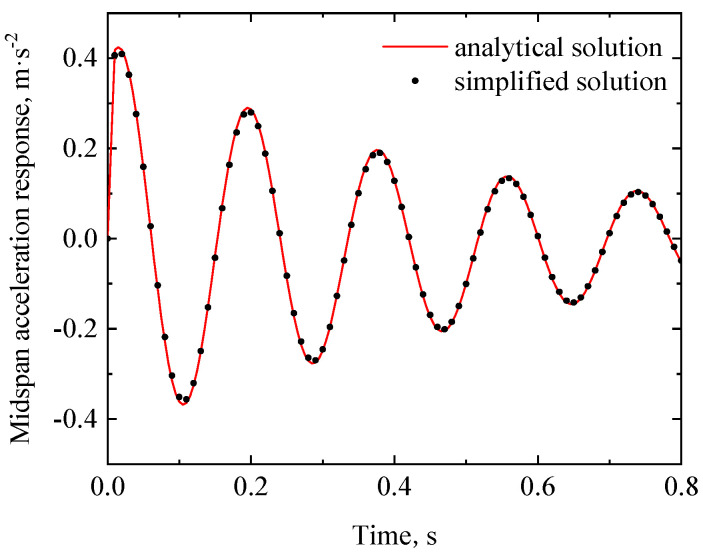
Curves of acceleration versus time for midspan of viscoelastic bearing RC beam.

In this numerical example, the approximate solution developed in Section 2.4 is validated by comparing it to the accurate solution developed in Section 2.2. All the parameters referred to are those used in Section 3.2. Figure 13 and Figure 14 show the midspan displacement and acceleration response, respectively. The approximate solution developed based on the simplified modal shape agrees perfectly with the accurate solution developed based on the modal shapes with complete information, indicating that the equation of motion developed based on the simplified model can be used for further analyses.

## 4. Discussion

This paper derives the dynamic response of viscoelastic-supported RC beams under moving loads, providing vital theoretical support for the optimal design of railway bridges (in terms of bearing stiffness, damping, and train speed) as well as structural safety assessments (focusing on vibration amplitudes). It offers a robust reference for both numerical simulation results and empirical predictions. Nevertheless, this model possesses certain limitations, as numerous random factors existing in practical engineering contexts were simplified, including wheel irregularity, track irregularity, and the hunting motion of wheelsets. Moving forward, based on this model, the resonance and vibration suppression mechanisms of viscoelastic-supported RC beams can be further explored, thereby enhancing the theoretical guidance for optimal bridge design and ensuring both ride comfort and safety.

## 5. Concluding Remarks

In this paper, the dynamic responses of the viscoelastic bearing RC beams subjected to a single load and equidistant moving loads have been investigated by an analytical approach. The following are some conclusions drawn from this study.

(1) This paper proposes a calculation method for the dynamic response of a viscoelastic-supported beam under moving loads. When the stiffness *k* and damping *c*_1_ are taken as ∞ and 0, this method can be degraded into a calculation method for the dynamic response of a rigidly supported beam under moving loads, and is in good agreement with the existing theoretical results of rigidly supported beams, proving the correctness of the proposed method in this paper. Subsequently, this paper also proposes an equivalent simplification method for viscoelastic-supported beams, which is proved to be effective after comparing with the analytical methods in Section 2.1 and Section 2.2.
(2) The impact of train speed, support stiffness, and damping on viscoelastic bearing RC beams is analyzed in this paper. Max displacement does not rise linearly with speed. The increase in stiffness at supports reduces displacement amplitude and raises fundamental frequency. A higher damping at supports enhances attenuation in free vibration. In practical engineering, we recommend controlling the train speed to avoid the dynamic amplification factor D reaching its peak. Additionally, by appropriately designing the stiffness of the support, we can control the fundamental frequency of the railway beam to prevent resonance in the viscoelastic-supported beam.
(3) The effects of stiffness and damping at the supports on the shape function of the viscoelastic bearing RC beam have been discussed. The damping at the supports hardly affects the shape function, but stiffness at the supports can affect it. Furthermore, based on the simplified shape function, the dynamic response of the viscoelastic bearing RC beam subjected to equidistant moving loads has been proposed by an analytical approach.

In free vibration, due to the effect of damping of supports, the response of RC beams in resonance may be significantly suppressed, but there remains a difference in the case of cancellation, in which the RC beams might still be adversely vibrated.

## Figures and Tables

**Table 1:** Main design parameters of the RC beam.

Parameter	Value
Length *L* (m)	32
Cross-sectional area *A* (m^2^)	8.97
Density *ρ* (kg/m^3^)	2500
Elasticity modulus *E* (GPa)	34.5
Area moment of inertia *I* (m^4^)	11.1
Damping ratio *ξ*	0.05

## Data Availability

The original contributions presented in the study are included in the article; further inquiries can be directed to the corresponding author.
